# Microscopic and Biomechanical Analysis of PEEK Interspinous Spacers for Spinal Fusion Applications

**DOI:** 10.3390/ma18030679

**Published:** 2025-02-04

**Authors:** Elliot Alonso Alcántara-Arreola, Aida Verónica Rodríguez-Tovas, José Alejandro Hernández-Benítez, Christopher René Torres-SanMiguel

**Affiliations:** 1 Sección de Estudios de Posgrado e Investigación, Escuela Superior de Ingeniería Mecánica y Eléctrica, Unidad Zacatenco, Instituto Politécnico Nacional, Mexico City 07700, Mexico; 2 Departamento de Microbiología, Escuela Nacional de Ciencias Biológicas, Instituto Politécnico Nacional, Prolongación Carpio, y Plan de Ayala s/n. Col, Casco de Santo Tomas, Mexico City 11340, Mexico

**Keywords:** biomechanical analysis, lumbar interbody fusion, experimental test, endoprosthesis

## Abstract

Spinal fusion is a surgical intervention used to join two or more vertebrae in the spine. An often-used method involves the placement of intervertebral spacers. They are commonly composed of biocompatible materials like polyetheretherketone. It has strength, longevity, and the capacity to interact harmoniously with the human body. Standardized mechanical tests were performed on two distinct implants to assess their biomechanical characteristics. The studies were conducted at a velocity of 2 mm/min. The stopping criteria were determined based on the loads sustained by the 50th percentile. Furthermore, the chemical composition of the implants was assessed using Raman spectroscopy. The implant created via subtractive manufacturing has a significant change in its elastic region at a force of 1300 N, and it begins subsidence when vertebrae are subjected to a load of 1500 N. The integration of microscopic characterization techniques with the mechanical analysis of prostheses in numerous case studies facilitates the biomechanical evaluation of implants.

## 1. Introduction

Spine fusion surgery is the fifth most-often performed operation in the United States each year. It is used to treat various cervical and lumbar spine illnesses that cause pain and disability [1]. In Sweden, 27,576 surgeries were conducted on 25,247 patients in 13 years of disc herniation procedures and related spinal degenerative disorders, a mean yearly surgical incidence of 24 per 100,000 individuals. The predominant demographic of patients was middle-aged, with a median age of 42 years, and males constituted 58% of the population [2]. In England, from 1999 to 2013, hospital admissions related to degenerative lumbar spine disorders and disc herniation escalated significantly, rising from 127 to 216 per 100,000 population, indicating a 1.7 rate increase. Women represented a greater proportion of hospital admissions, with their rates rising at double the pace of men [3]. Over 15 years in Norway, the incidence of lumbar spine surgeries increased from 77.8 per 100,000 in 1999 to 119.9 per 100,000 in 2013, reflecting a 54% rise. The majority of procedures were performed on patients aged 40 to 59 years [4].

There are various surgical methods for fusion. One commonly employed technique involves placing an intervertebral spacer (ISP) or lumbar cage (LC) between the spinal vertebrae. These devices relieve pressure on the nerves, enhance spinal alignment, and improve both mechanical stability and the fusion rate after surgery [5,6,7]. The ultimate success of interbody fusion depends on the full integration of bone tissue between the LC and vertebral endplates. To enhance the effectiveness of fusion, many materials and designs for interbody cages have been created [8,9]. Interbody fusion following cage implantation involves a complicated interaction of biological and mechanical elements [10,11,12]. Interbody cages are often made of either polyetheretherketone (PEEK) or titanium (Ti) [13]. PEEK is a high-performance polymer that possesses outstanding mechanical, thermal, and electrical capabilities, as well as excellent chemical resistance [14]. PEEK is a suitable material for load-bearing implants with complex geometries due to its biocompatibility and mechanical properties, which are similar to those of cortical bone [15]. The most recent studies [16,17,18] indicate that PEEK’s cell is an orthorhombic crystalline unit. The positions of more substantial peaks corresponding to plan (110), (111), (200), and (211) are used to identify the a- and b-axis in the radial direction and the c-axis in the lamellae thickness direction. The c-axis of the unit cell coincides with 2/3 of the elementary pattern. The crystallographic plane (110) appears to be a preferential growth plane [19,20].

Several methods can be used to assess the crystallinity of polymers, including wide-angle X-ray diffraction (WAXRD), small-angle X-ray diffraction (SAXS), differential scanning calorimetry (DSC), Fourier transform infrared (FTIR) spectroscopy, and Raman spectroscopy (RS) [21,22]. RS can be conducted using reflection geometry, which offers a high resolution comparable to optical microscopy. This technique can achieve sub-µm resolution and has the potential to analyze the local vibrational spectrum. Typically, the change in frequency of the main vibration modes that involve one or more covalent chemical bonds (known as intramolecular modes) is employed to assess the crystalline nature of a substance [23].

Recently, extensive studies have been conducted analyzing PEEK’s chemical behavior by Raman spectroscopy [24,25,26,27,28,29,30] or have performed mechanical evaluations for PEEK with mechanical proofs or with the finite element method (FEM). Masafumi et al. [31] assess the effectiveness of plasma-sprayed Ti coated on bone formation at the surface of bone implants by directly comparing coated and uncoated PEEK cages placed in the same intervertebral area. They conclude that radiographic indications observed surrounding the coated PEEK cage indicate the presence of bone ongrowth, as evidenced by the visible vertebral cancellous condensation around the cage. This contrasts with the uncoated PEEK cage, which does not show the same level of bone ongrowth. Yanfei et al. [32] developed a minimally invasive expandable fusion device that can decrease the occurrence of nerve damage caused by medical intervention and minimize damage to the endplate during oblique lateral interbody fusion (OLIF). They employ finite element analysis to assess the biomechanical stability of the newly developed expandable fusion device after its placement in intervertebral space. They concluded that bidirectional expandable cage (BEC) implanted models had a higher stiffness and better posterior fixation stress distribution than the OLIF models. The BEC models had a bit higher endplate and cage stress maximum values than the OLIF models, but they were still clinically acceptable. Kazunari et al. [33] generated a vertebrae’s FEM and performed a biomechanical stress analysis to compare the single-banana cage with the double-banana-shaped cage. They examined the efficacy of transforaminal lumbar interbody fusion (TLIF) employing two banana-shaped cages in achieving favorable clinical outcomes. The study’s findings indicate that TLIF utilizing double-banana-shaped cages resulted in favorable clinical outcomes and reduced cage subsidence. The study also emphasized the significance of cage placement and the dimensions of the contact surface area in minimizing cage subsidence. Prashant et al. [34] evaluate and compare the stress shielding caused by different types of traditional and modified additive-produced porous materials (PEEK, CFR-PEEK, Ti) that are utilized in an LC. A finite element analysis was conducted to investigate the effects of altering the design and materials of spinal cages. The findings indicated that a hybrid LC transmits a greater amount of stress to the neighboring vertebrae compared to the other design configurations when subjected to uniaxial compression. The hybrid cage demonstrated superior efficacy in mitigating the stress shielding phenomenon.

This study aims to analyze how the spacers distribute mechanical loads across the lumbar vertebrae (biomechanic properties) and material’s biocompatibility based on the stiffness, microstructure, and chemical composition of the PEEK-OPTIMA (PO) (first medical-grade PEEK utilized in spinal fusion procedures), compared to a PEEK made using subtractive manufacturing (SM) and additive manufacturing (OM).

## 2. Materials and Methods

**Figure 1:**
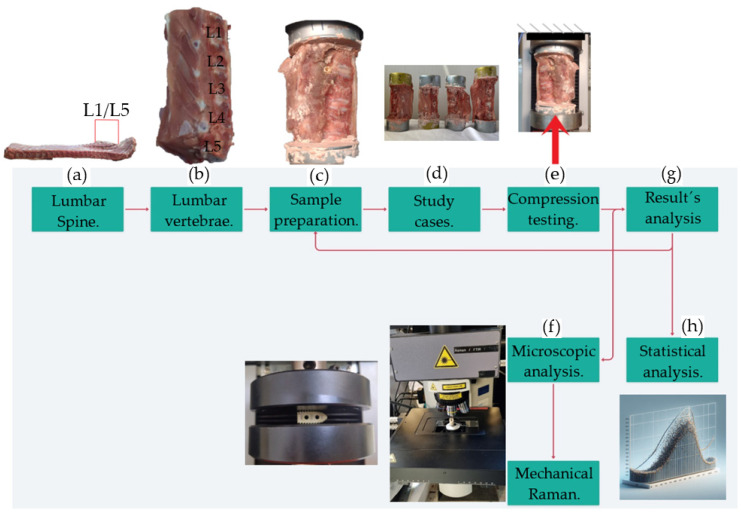
Methodology. (**a**) Location of the lumbar region to remove the vertebrae. (**b**) Lumbar vertebrae. (**c**) Sample fixation to cans with dental cast. (**d**) PO and PEEK LC. (**e**) Compression test diagram. (**f**) Raman spectroscopy and compression test experiments. (**g**) Evaluation of the obtained results. (**h**) Polynomial regression.

Figure 1 summarizes the methodology. Firstly (Figure 1a), vertebrae L1–L5 are removed from the swine’s lumbar region. Figure 1b shows the extracted lumbar region. Medical instrumentation (pedicle probe, pedicle awl, pedicle screwdriver, rod bender, rongeurs, drill guides, surgical retractors) is required to insert the LC into vertebrae. The drill hole should be 2 mm smaller than the implant dimensions to ensure proper tightening. After, the sample is fixed into cans with a dental cast (Figure 1c). The experimental design evaluated the biomechanical behavior of sixteen study cases (Figure 1d). Compression (Figure 1e) tests are performed to determine the system’s biomechanical behavior. Subsequently, Raman spectroscopy and biocompatibility analysis are developed in LC (Figure 1f). Finally, a polynomial regression is applied to the results to indicate if the experiments were carried out correctly or if the technique used to obtain the specimens should be corrected (Figure 1f,h).

### 2.1. Device Description

**Figure 2:**
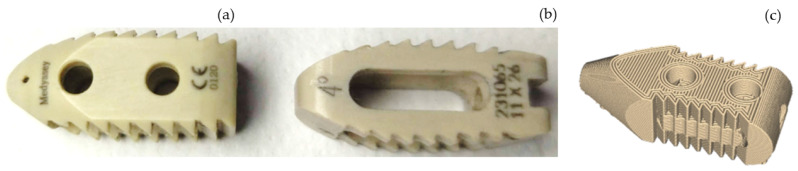
PEEK lumbar cages: (**a**) PEEK Optima cage. (**b**) SM PEEK cage. (**c**) OM PEEK cage.

The PO LC (Figure 2a) is fixed intersomatically between the lumbar spinous apophyses areas. The LC is a cannulated component that enables easy insertion and positioning within the spine. The interspinous cage is surgically implanted between the spines to ensure a sufficient gap between the elements. Teeth anchor the cage. Due to PEEK’s mechanical behavior, the graft material experiences more significant stress and is expected to undergo solid bone remodeling in accordance with Wolff’s law [35]. Figure 2b shows the geometry of the SM implant. The OM PEEK (Figure 2c) optimal tensile properties are achieved using the following parameters: a printing speed of 60 mm/s, a layer thickness of 0.2 mm, a printing temperature of 370 °C, and a filling ratio of 40% [36]. Using a nozzle diameter of 0.4 mm, a nozzle temperature of 430 °C, and a printing speed of 5 mm/s is advantageous for achieving the most compact samples and, thus, the greatest bending strength. The optimal compression qualities were attained using a 0.6 mm nozzle, with minimal impact from the other parameters [37]. Additionally, increasing the melt pressure aids in minimizing the surface imperfections of an extruded filament [38].

Spacers PO and SM (Figure 2a,b) are used to perform the compression tests. An OM (Figure 2c) spacer is used to compare fungal biofilm formation with respect to the implants made in cold casting.

An LC can be inserted into the intervertebral disc space using a conventional anterior cervical discectomy and fusion surgical approach, which is comparable to the method used for placing other cervical cages [39].

### 2.2. Sample and LC Testing

Sixteen swine spine segments from the lumbar area were utilized for the experiments. A biomechanical swine model was used due to its similarity to humans in terms of femoral dimensions, areas, lamellar bone structures, bone regeneration mechanisms, bone mineral density, and concentration [40]. Evidence has demonstrated that the swine lumbar spine can serve as a viable animal model for studying the biomechanics of the human lumbar spine [41].

**Figure 3:**
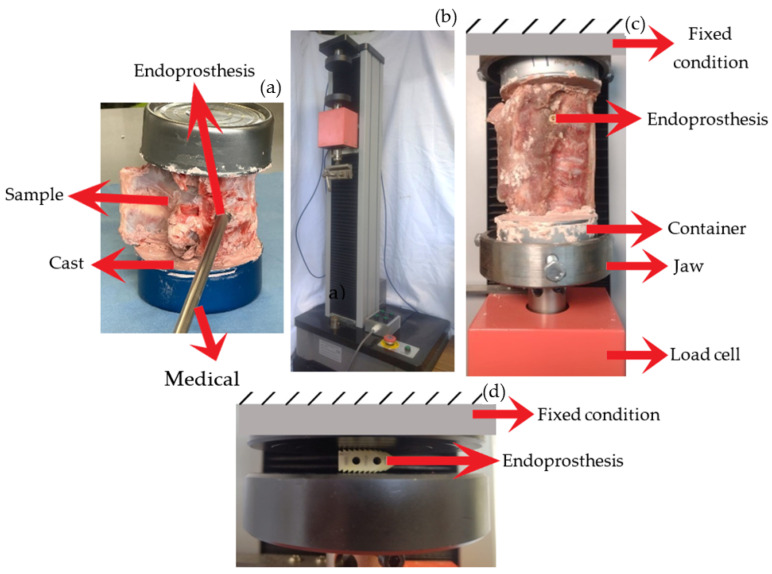
Scheme of the tests performed. (**a**) Endoprosthesis fit. (**b**) WDW-5 machine for compression tests. Load accuracy ≤ ±1%. Displacement resolution 0.01 mm. (**c**) Free body diagram, P is the load. (**d**) Illustration depicting the studies conducted at the LC.

Sixteen swine corpses were stored at a temperature of −20 °C and thawed 8 h before testing. Muscle tissue was extracted from the vertebrae, with special attention given to preserving spinal ligaments and facet joints. Prior to conducting mechanical testing, the specimens were thawed at an ambient temperature of 20 °C. Afterwards, a pilot hole was drilled between the L3 and L4 vertebrae. The diameter of the hole was 2 mm less than that of the LC, ensuring adequate tightening and preventing any movement of the prosthesis. The LC was positioned using the requisite medical equipment (Figure 3a). The lower and upper parts of the vertebrae were secured using a dental cast in containers, which were positioned within the machine’s clamps. The device shown in Figure 3b is “WDW-5 Computer Control Electronic Universal Testing Machine”. The compression tests were conducted using ASTM E-9 [42] as a reference, with the necessary modifications made. The load was exerted on the lower portion of the vertebra (Figure 3c), while the higher portion was fixed. The force was applied at a rate of 2 mm/min. The tests were conducted with a load that corresponds to the average strength of a 50th-percentile human.

The spacers conducted compression testing (Figure 3d) according to ISO 604 [43]. The load cell exerted the force at a velocity of 2 mm/min. This is equivalent to a strain rate of 0.0002 s^−1^, a quasi-static condition [44]. The upper section of the machine limited the motion, while the lower section exerted a compressive load. WD 40 lubricant was applied to the compression plates to minimize the coefficient of friction and prevent any impact on the results. The stop criteria for terminating the experiments were established on the fact that in the Mexican population, the lumbar area in a normal standing posture absorbs two-thirds of the weight [45] (500 N). The loads exerted on the lumbar vertebrae while walking can reach up to 2 to 2.5 times the individual’s body weight. The importance of these weights is highest at the toe-off phase and becomes more significant as walking speed increases. The lumbar area bears a load that is four times the weight of the body in some exercises, such as rowing [46]. Table 1 summarizes the conditions to perform the experiments.

It is advisable to initially insert the LC into the sample and then secure it to the cans. The Max Test program was utilized to quantify the machine’s jaw displacement and the load cell’s capacity. It is crucial to note that the program estimates the displacement of the load cell for all conducted experiments. Additionally, according to the experimental setup, the displacement of the load cell is equivalent to the deformation of the system.

### 2.3. Raman Spectroscopy

The chemical composition and crystalline structure of the PEEK LC were analyzed using Raman spectroscopy. The method is highly effective in the examination of the material’s vibrational modes, which are directly associated with mechanical properties such as rigidity and load distribution capabilities. Vibrational modes, or phonons, denote the collective vibrations of atoms inside a material’s structure. These oscillations are intrinsically linked to the material’s bonding forces and structural configuration, rendering them sensitive indicators of mechanical qualities such as stiffness, elasticity, and strain response. The correlation between vibrational modes and mechanical properties is frequently investigated by techniques such as Raman spectroscopy, which enables the precise observation of variations in vibrational frequencies. Analyzing these shifts allows for the inference of significant mechanical characteristics [47,48]. It is imperative to comprehend these aspects in order to assess the material’s response to compressive loads. Raman spectroscopy offers supplementary chemical and structural information to mechanical testing results. By establishing a correlation between microstructural characteristics and observed mechanical behavior, we can gain a more comprehensive understanding of the material’s capabilities and constraints. For instance, the identification of changes in crystallinity through Raman spectroscopy can contribute to the elucidation of variations in deformation behavior, elasticity, or rigidity that are observed during experimental testing.

The instrumentation utilized for the examination was a LabRam HR 800 Horiba Jovin Yvon (HORIBA, Kyoto, Japan), a Confocal Microscope Olimpus BX41 (Microscope Central, Feasterville, PA, USA), and a spectrometer FTIR llliminatIR IR2 model with a source Argon laser that emits at 514 nm. The analyses were performed with an objective ×100 and a numerical aperture of 0.9. The spot diameter, axial resolution, and spectral resolution were 858 nm, 2.8 μm, and 0.3 cm^−1^, respectively. The confocal aperture had a diameter of 30–35 μm, and the holographic network consisted of 600 lines/mm. In this investigation, ten spectra were obtained by scanning the grating over a wide spectral range (from 100 cm^−1^ to 1800 cm^−1^) in ten separate locations of the sample. Each spectrum was recorded with an exposure length of 10 s. The surface of the PEEK LC was 80 μm × 80 μm. Prior to testing, the specimens underwent cleaning using a solution consisting of 70% alcohol and 30% water. Table 2 helps to identify the bonds in PEEK composition. The vibration modes were as follows: ϒ: out-of-plane shear strain; δ: in-plane shear strain; ν: stretching; vw: extremely low, measuring less than 2%; w: indicating a level of intensity that falls between 2% and 5%; m: representing a moderate intensity, ranging from 5% to 15%; s: signifying a high intensity, measuring between 15% and 50%; vs: indicating an exceptionally high intensity, surpassing 50% [25].

### 2.4. Fungal Biofilm Formation on Implants

The PEEK devices (SM, PO, and OM) were sterilized using an autoclave at 121 °C for 15 min prior to use [49]. The fungal biofilm was established as previously described on the PEEK devices placed at the bottom of wells in a polystyrene plate and incubated at 37 °C for 24 h, including a sterility control. After incubation, the supernatant was removed, and the biofilm was rinsed twice with 1X PBS. Fixation was performed by covering the samples with 2.5% glutaraldehyde (Sigma-Aldrich, St. Louis, MO, USA) for 2 h, followed by 1% osmium tetroxide (Sigma-Aldrich, St. Louis, MO, USA) for 2 h, and washing twice with 1X PBS after each fixation step. Then, the samples were dehydrated with increasing concentrations of ethanol (10–90%), reaching absolute ethanol, and subsequently dried to the critical point with 1,1,1,3,3,3-hexamethyldisilazane (Electron Microscopy Sciences, Washington, PA, USA). Finally, the samples were coated with gold and observed using a scanning electron microscope (SEM) (JSM-7800F, JEOL Ltd., Tokyo, Japan) at the High-Resolution Scanning Electron Microscopy Laboratory of the Nonsciences, Micro, and Nanotechnologies Centre at the National Polytechnic Institute (CNMN-IPN), Mexico City.

## 3. Results

**Figure 4:**
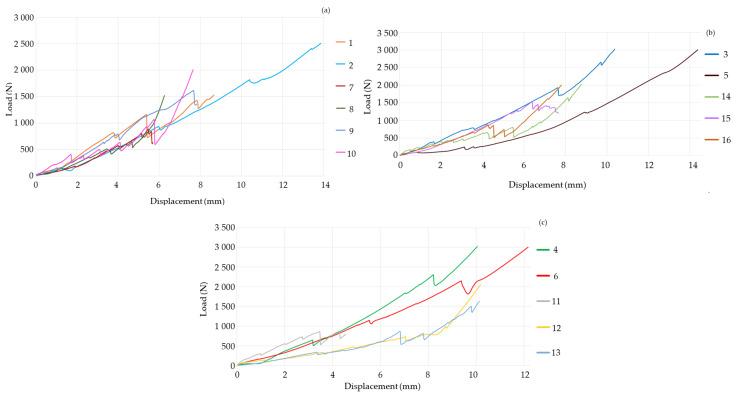
Experimental results. Load–displacement. (**a**) Graph without LC. The system exhibits a failure range of 635 N to 1164 N, with corresponding deformations between 4.668 mm and 5.98 mm. These results demonstrate the baseline behavior of vertebrae under compressive loads. (**b**) Graph with PO LC. Samples displayed a broader load-bearing capacity, with peak forces from 868 N to 1927 N and corresponding deformations from 4.27 mm to 7.61 mm. The PO implants showed an efficient load distribution and higher stiffness compared to samples without LC. (**c**) Graph with SM LC. The system has a force capacity from 855 N to 1841 N. Subsidence behavior is performed around 2297 N, indicating localized deformation and reduced load distribution efficiency compared to PO implants.

Figure 4 presents a concise overview of the acquired data. Samples 1 to 6 utilized the L1–L5 lumbosacral segments for the studies. The remaining samples were conducted using vertebral units consisting of L4–L5.

Figure 4a displays the experimental outcomes of the research cases conducted in the absence of LC. The complete vertebral unit systems (Samples 1 and 2) experience failure at a range of 935 N to 1164 N. The discrepancy arises from the toe effect that originated in experiment 1. By modifying the behavior of specimen 1 to account for the toe effect, the load at which the material fails is determined to be 976 N. The deformation values for experiments 1 and 2 are 5.384 mm and 5.98 mm, respectively. Experiments 7, 8, 9, and 10 exhibit failure within the load range of 781 to 803 N, in combination with deformations ranging from 4.02 to 5.135 mm. The disparity of 173 N in the failure loads can be attributed to the presence of additional vertebral segments and intervertebral discs in specimens 1 and 2. With an increased volume, they possess the capacity to store a greater amount of energy and endure more substantial loads. Nevertheless, the graph demonstrates that the stiffness remains consistent across all specimens, independent of the number of lumbar vertebrae in the system.

The results of the experiments with the PO spacer are shown in Figure 4b. Experiment 5 exhibits a reduced slope. This is because the vertebrae were smaller than the others. The measurements are impacted by the distribution of the load across a reduced cross-sectional area. This experiment is eliminated to ensure that it does not affect the sampling of the other experiments. Specimen 3 can hold a force of 1924 Newtons and experiences a deformation of 7.615 mm. Experiments 14, 15, and 16 exhibit a force range of 800 to 867 N, accompanied by deformations ranging from 4.269 mm to 5.468 mm. The discrepancy in outcomes arises from the fact that specimen 3 possesses a more significant number of elements onto which the load can be evenly distributed. Nevertheless, it has been noted that despite the number of vertebrae in the system, the slopes remain similar. When comparing with the tests shown in Figure 4a it is observed that there is an increase in stiffness.

The results of the tests conducted using the LC SM appear in Figure 4c. The stiffness of tests 12 and 13 is approximately 90.66 N/mm. Experiments 4, 6, and 11 have a stiffness ranging from 233 to 250 N/mm. The variation could be attributed to the manipulation of the specimen before its acquisition. The consequence is the experience of impact loads that undermine the mechanical efficiency during the experiments.

**Figure 5:**
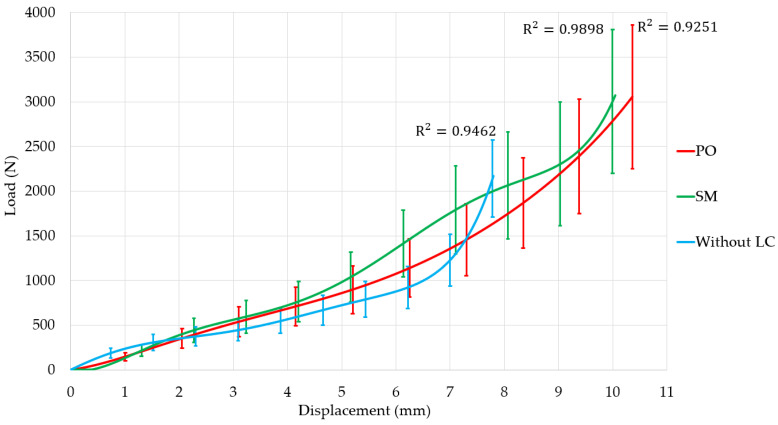
Experimental polynomial regressions. They are obtained from average data.

Figure 5 displays a statistical analysis that compares the results of the studies using polynomial regressions.

Specimens 5, 12, and 13 were excluded due to their disparity. The coefficient of determination (R^2^) exceeds 90% in all instances, showing high consistency in the execution of the experiments. The slope is an indicator of the system’s rigidity. The use of spacers increases the rigidity of the system since the prostheses contribute to enhanced stability. The mechanical characteristics of the graphs suggest that the PO LC exhibits superior biomechanical performance due to its smooth curve, which suggests more efficient load distribution within the system. The SM LC enhances the system stiffness, but it does not distribute loads as efficiently, as evidenced by the transitions in the green function. The increased rigidity of the system results in reduced deformation compared to the PO, posing a risk of device fracture.

The rising error bars in Figure 5 emphasize the growing variation in mechanical performance at elevated displacement levels, mainly attributable to material anisotropy, microstructural defects, and the nonlinear behavior of the spacers during the specimen’s plastic deformation. These changes underscore the necessity of constant manufacturing and stringent quality control, particularly for applications necessitating reliable mechanical performance.

**Figure 6:**
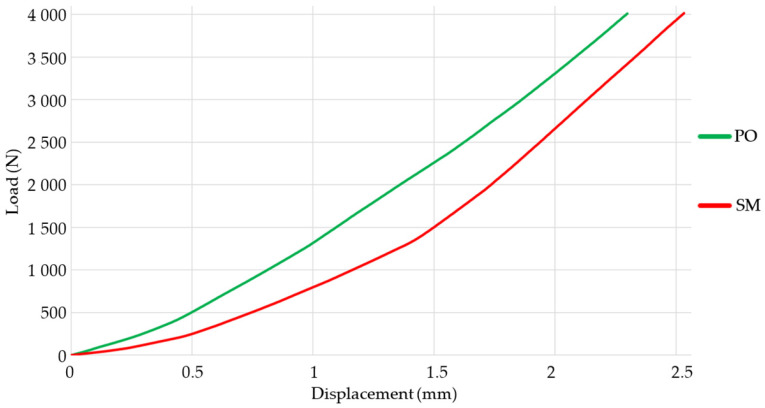
Summary of compression test results for interspinous spacers.

The compression test results of the spacers can be seen in Figure 6. The mechanical behavior suggests that the PO spacer is more rigid than the SM spacer. Nevertheless, the graph clearly shows that the SM spacer has two distinct slopes, with the transition occurring at around a load of 1300 N. While the spacer itself does not fracture, the presence of a change in the stiffness suggests that the loads are distributed uniformly. The alteration in the slopes suggests that the material undergoes two phases of elastic deformation. This signifies the existence of distinct microstructures or phases inside the material that exhibit varying responses to the applied load. The initial slope represents the elastic deformation phase, during which the material’s crystalline structures are crushed in a reversible manner. Slope two represents a subsequent phase of elastic deformation, potentially linked to an additional microstructural alteration or reorientation of the material’s phases. This is related to Figure 5, where a curve with distinct variations is observed when the spacer is connected to vertebrae.

Raman spectroscopy tells us a lot about how the methods used to make things change the microstructure of materials, which in turn changes how well they work in compression tests. For example, using materials with higher crystallinity, like those in PO spacers, can make them stiffer and lower the chance that they will break or subsidence, which makes them more reliable for implant designs.

**Figure 7:**
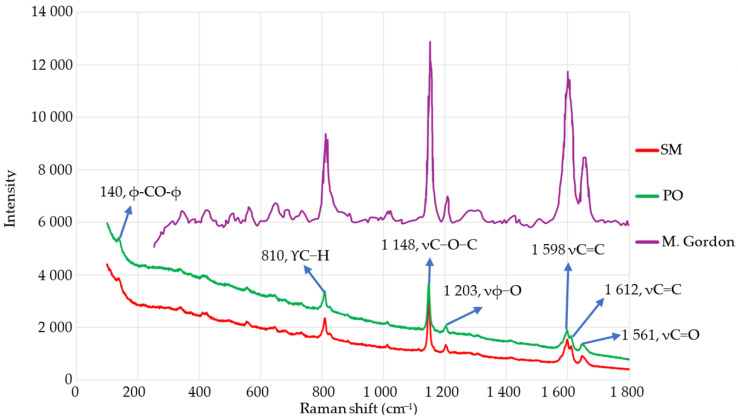
PEEK samples’ spectrum.

Figure 7 displays the outcomes of Raman spectroscopy, comparing the findings of the American and Indian samples with those of M. Gardon et al. [50]. The graph demonstrates minimal change in amplitudes along the X-axis, confirming that the investigated material is PEEK. The disparities in the magnitudes of the amplitudes between the LC and the PEEK they are being compared to are caused by the objectives employed. An objective with a magnification of 100× was utilized in our study, but Gardon employed an aim with a higher magnification lens (20×).

The Raman spectrum is separated into two distinct parts based on frequency: the low-frequency region (100–200 cm^−1^) and the medium-frequency region (200–1800 cm^−1^). Yamaguchi et al. [21] obtained spectral data of PEEK in the lower frequency range. A peak at 135 cm^−1^ was found. At a frequency range of around 130–145 cm^−1^, the researchers identified the rotational movement of the ketone group and its connection to the benzene rings. At approximately 175–190 cm^−1^, they identified the rotational motion occurring around the ketone groups. The observed behavior closely resembles the pattern reported in our experiment. The PEEK is observed with a frequency of 139 cm^−1^. Delbe et al. [28] propose that the middle region can be further divided into the following:

- The frequencies of 200–1020 cm^−1^ correspond to the out-of-plane C-H deformation of the hydrogen atoms that are bonded to the aromatic rings, known as γC−H. This region exhibits a multitude of modes ranging from strong to extremely weak intensity, which corresponds to deformations of the C-H bond in the phenyl ring that occur out-of-plane.
- The frequencies of 1020 and 1200 cm^−1^ correspond to the in-plane deformation of the C-H bonds linked to the aromatic rings, as well as the stretching of the C-O-C bonds. In PEEK, the C-O-C stretching mode is utilized for spectral normalization due to its lower sensitivity to microstructural differences compared to other vibration modes.
- Within the range of 1200 to 1540 cm^−1^, there is a stretching of C-O or C-O-C bonds, specifically vC−O or νC−O−C. In this region, there is a peak at 1203 cm^−1^ that corresponds to the antisymmetric version of the strong C-O-C stretching found in the prior zone.
- The stretching vibration of the C=C ring, vC=C, occurs within the range of 1540 to 1635 cm^−1^. Two quite pronounced modes are observed in the spectra at around 1598 and 1612 cm^−1^. Briscoe et al. [51] attribute the 1595 and the peak at 1607 cm^−1^ to the vibration of the phenyl ring. The sample’s modes are dependent on the laser’s orientation and polarization [52].
- The stretching of the carbonyl C=O bond in the ketone group, denoted as vC=O, occurs between 1635 and 1700 cm^−1^.

The coordinates of the material’s peaks and behaviors agree with the information provided in Table 2, which presents a detailed breakdown of the material’s chemical behavior.

The study found that the PEEK materials had different crystallinity and molecular structures. Different molecular orders were shown by peaks connected to different vibrational modes like C-O-C or C=C bond stretching. The force–displacement graphs showed that spacers with higher crystallinity, like those made with the PO LC, were stiffer. This means that a structure with more crystallinity makes it more resistant and lets the load distribute further uniformly.

The SM LC, on the other hand, showed signs that its structure might be flawed or inconsistent, such as having less crystallinity or amorphous phases. These spacers also tended to subsidence under heavier loads (about 1500 N), which suggests that they cannot support and distribute loads as well because of flaws or less crystallinity inside them. This could damage the tissues around them. Different vibrational modes also show how materials act when they are in their elastic state. Peaks corresponding to C-O-C and C=C bond stretching show that the polymer can handle deformations that can be undone. The force–displacement curves for the PO spacers were smoother and more uniform, which suggests that they had more initial elasticity before changing into plastic deformation. This is in line with the fact that Raman analysis showed that they were crystallized.

Scanning electron microscopy was utilized to determine whether and how *C. albicans* ATCC 10231 forms biofilms on PEEK devices.

**Figure 8:**
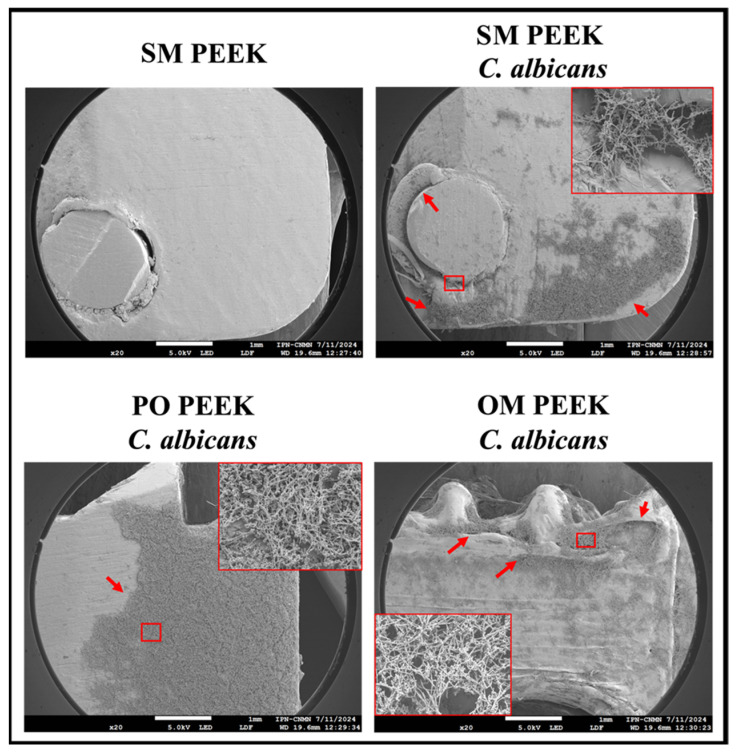
*Candida albicans* biofilm formation on PEEK lumbar cages. The ability of *C. albicans* ATCC 10231 to form biofilms on PEEK devices was analyzed. Extensive colonization (indicated by red arrows) and multilayering of yeast hyphal networks (as seen in higher magnification boxes [500×]) were observed on each device, facilitated by the roughness and cracks in the surface structure of the PEEK material.

Figure 8 illustrates the development of C. albicans biofilms on the different intervertebral spacers: SM PEEK, PO PEEK, and OM PEEK. Initially, the surface of the uninoculated SM PEEK is shown, characterized by a smooth appearance with gaps corresponding to the locations of rivets. Further, on the surface of the inoculated SM PEEK device, dispersed yeast colonization is evident. In the gaps between the device and the metal rivet, the deposition of blastoconidia and the development of hyphal and pseudohyphal networks are observable, as highlighted in the red box.

**Figure 9:**
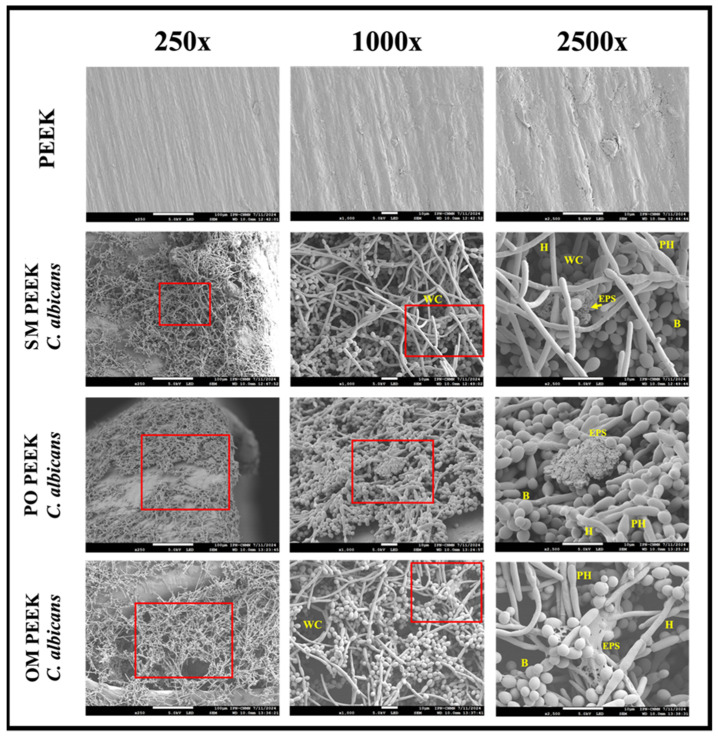
Morphology of the C. albicans biofilm on PEEK devices. The fungal biofilm developed on the different LCs was studied in detail. In all cases, the presence of clusters of blastoconidia was evident, primarily located in the basal zone of the biofilm, supporting intertwined hyphae and pseudohyphae that give rise to a three-dimensional arrangement. Additionally, the production of exopolymeric substances (EPSs) that cover the cells and accumulate in the intercellular spaces was observed. H = hypha, PH = pseudohyphae, B = blastoconidia, EPS = exopolymeric substance, WC = water channels.

Figure 9 also shows the production of extracellular polymeric substances (EPSs). In PO PEEK extensive colonization is apparent on the surface, with clusters of blastoconidia adhering to the substrate and supporting hyphal and pseudohyphal networks, as revealed in the red box. Figure 9 further illustrates abundant pseudohyphae and EPS in the intercellular spaces. Finally, in OM PEEK, the surface exhibits increased roughness and irregularities, which facilitate yeast proliferation and adhesion, ultimately allowing the development of biofilm. This is evident in areas with substantial growth, where biofilm begins to develop, as shown in the red box. These sites display deeper biofilm layers, as seen in Figure 9, with blastoconidia at the bottom of cracks, numerous hyphal and pseudohyphal networks forming water channels, and outlining the biofilm’s three-dimensional structure. An EPS covers and connects the cells, enhancing the overall biofilm structure.

## 4. Discussion

This study involved conducting compression testing on lumbar cage implants. The biomechanical properties of the LC have been evaluated in relation to the vertebrae. The mechanical properties of the spacers were also investigated. Stiffness serves as a good metric for evaluating these circumstances [48,53,54,55]. Load–displacement curves gave enough data to indicate patterns in stiffness, load distribution, and deformation, which directly addressed the primary goal of comparing spacer performance. These curves provide a direct picture of the mechanical reaction to applied stress and are critical for understanding real phenomena, including subsidence, stiffness changes, and elastic deformation phases. Additionally, a chemical investigation using Raman spectroscopy was conducted to explain its crystalline structure.

The experiments have the next limitations. Firstly, the geometry of the vertebrae prevents the samples from being aligned longitudinally with the application of the load. Secondly, the eccentricity of the samples provides a bending moment that the program is unable to measure. The sample comprises hard and soft tissue, metallic cans, dental casts, and intervertebral discs. Numerous materials exist; however, the software only quantifies the applied load and the deformation of the overall system. The research does not reproduce the LC’s interaction with the whole range of forces (moments) produced by the body’s movement, only considering the compression loads.

Nevertheless, the outcomes of the experiment remain unaffected by this. If more forces are introduced, the behavior seen will be comparable to that described in the Results section. Furthermore, the objective of this study is to examine the crystalline structure of the implants to gain a deeper comprehension of their biomechanical capabilities.

The samples lacked a pedicle screw fixation mechanism. Multiple researchers have documented the utilization of posterior internal fixation equipment to preserve stability at the operated level [56,57]. However, they do not assess the biomechanical properties of materials. Many researchers [32,58,59] have performed biomechanical studies of LCs using the finite element approach. One of their case studies is evaluating the efficacy of a cage without a pedicle screw. They determine that pedicle screw fixations are superior in reducing both stresses and the range of movements (ROMs). The von Mises stresses and the ROMs during the simulation of the implant alone do not surpass the critical thresholds for the material.

Additionally, the fixation method decreases the number of examined parameters, but they do not analyze the mechanical forces resulting from the interaction between the materials. If stress is reduced, the bone will lose its capacity to store energy and provide load-bearing support. Furthermore, the authors suggest that the system exhibits good stability even in the absence of the pedicle screw fixation system.

During the test, the temperature and the speed at which the load is applied were monitored. The machine was calibrated with ISO standards. Figure 6 shows an R2 higher than 90%. This suggests that the experiments have a high level of reproducibility.

**Figure 10:**
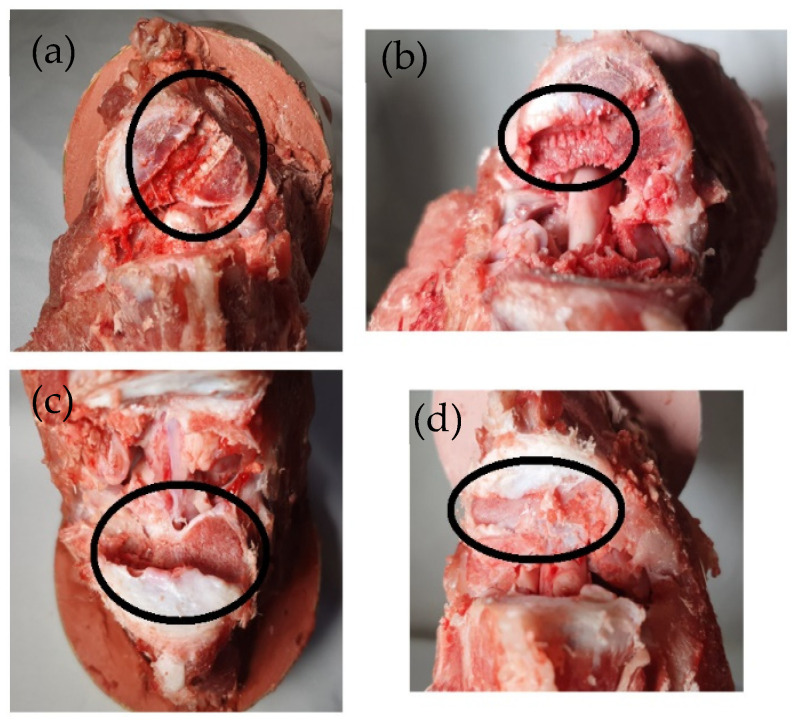
Interplay between vertebral and LC. (**a**) Sample 4, implant teeth are subsidence in the system. (**b**) Sample 11, the area where the implant teeth are subsidence is indicated. (**c**) Sample 3, the loads are evenly distributed, and the implant is not subsidence. (**d**) Sample 15, the implants are not subsidence.

Figure 10 illustrates the biomechanical response of the implants in relation to the bone. Figure 10a,b show how the SM spacer was inserted into the vertebrae. Due to the inability to evenly distribute the load, the forces become focused at specific spots, resulting in the LC becoming a subsidence process. This happens at around 1500 N. Figure 10c,d exhibit the absence of marks left by the PO LC. The visible space is created when the device is inserted. However, the figures emphasize the lack of tooth marks. Figure 5 demonstrates that the prosthesis effectively distributes forces by accommodating bigger displacements, resulting in improved biomechanical performance.

Figure 7 demonstrates that the PO implant graph exhibits more height compared to the SM graph, suggesting a bigger fluorescence intensity for the PO implant. Consequently, this suggests that the crystalline characteristics of the PO implant are more refined. The data are consistent when comparing the chemical composition, fluorescence, crystallinity, and biomechanical performance of the implants. The presence of voids and defects in the SM implant material is indicated by its crystalline characteristics, resulting in the observed mechanical behavior.

Polynomial regression was performed to model the biomechanical response trends and confirm the reproducibility of the experiments by high coefficients of determination. This instills confidence in the consistency of the data across experiments. Although regression lines were central to our analysis, the high R^2^ values underpin a good fit to the observed data and indirectly support the reliability of the measurements. Further reinforcement is that the experimental conditions were closely controlled to meet the requirements for ISO and ASTM standards on compression testing, which makes such results more valid and repeatable. No usual statistical tests, such as ANOVA and *t*-tests, have been applied in this work. Still, we would like to underline that the purpose of the study was the assessment and comparison of biomechanical trends and not the detection of statistically significant differences between groups based on hypothesis testing. Figure 5 displays error bars that indicate the variability in the measurements of force–displacement behavior for the PEEK interspinous spacers.

Variations occur because of slight inconsistencies in the material properties, manufacturing precision, or testing conditions. Larger error bars are frequently noted in SM spacers, attributable to the inherent variability in manufacturing processes. SM techniques can induce variations in microstructure, leading to inconsistent mechanical performance among samples. Variations in sample geometry and microstructure influence force distribution and deformation behavior under load, leading to variability. During the elastic phase, error bars are generally reduced due to the more predictable nature of the material’s response. During the plastic deformation phase, the error bars increase due to potential microstructural failures, variations in material behavior, or differences in the rate of deformation. At elevated displacement levels, the internal structure of the material, including microvoids, imperfections, or inconsistencies in the fabrication process, increasingly affects the material’s behavior. At more significant displacements, stress concentrations develop at locations within the spacer, which may differ between samples due to minor geometric variations or imperfections. Stress concentrations lead to uneven load redistribution, which results in variability in material deformation under increasing force. Reduced error bars in PEEK Optima indicate that the material exhibits reliable performance.

Lallemant et al. [60] conducted a tensile test on soft tissue. They reported a tendency for error bars to increase as the material changed from the elastic to the plastic region. A behavior analogous to that is shown in Figure 5.

Raman spectroscopy was utilized to examine the molecular interactions and structural characteristics of PEEK, focusing on the vibrational modes linked to significant chemical bonds, including C-O-C and C=C. The vibrational modes are directly associated with the degree of crystalline, which consequently relates to the mechanical properties [23]. The C-O-C bond, typical of the ether linkages in PEEK, enhances the flexibility and durability of the polymer chain. Fluctuations in the intensity or location of the C-O-C vibrational modes offer insights into the influence of strain or fabrication techniques on these bonds, potentially modifying the material’s elastic or plastic deformation characteristics. This agrees with Kong et al. [61], who demonstrate how Raman spectroscopy tracks the deformation of PEEK, particularly C-O-C and C=C bonds, correlating these with mechanical stresses.

The structural, biomechanical, and biocompatibility characteristics of PEEK have garnered significant attention for their biomedical applications. However, the latent risk of infection when used as an implant may limit the use [62,63,64,65]. Microbial growth on medical devices represents a significant risk to patients and can influence whether the body accepts or rejects the implant [66]. This concern is amplified when biofilm development is involved, as it can lead to chronic or recurrent treatment-resistant infections with lower therapeutic success rates [67,68]. In this study, we evaluated how Candida albicans colonize and form biofilm on different LCs. This fungus is a major fungal opportunistic pathogen associated with invasive infections, capable of forming biofilm, and is an important causal agent of nosocomial infections and infections associated with medical devices [68,69,70]. Figure 9 shows extensive yeast colonization on the surface of the intervertebral spacers, giving rise to distinctive biofilm structures. This outcome is expected, given that, despite its favorable traits as a biomaterial, PEEK lacks antimicrobial properties. Various strategies have been explored to address this issue, including chemical modification or functionalization with antimicrobial molecules [30,71,72,73,74], which could add the ability to inhibit microbial development to its already promising attributes. Therefore, further studies are proposed to address this issue. Figure 10 provides details of the mature biofilm of C. albicans formed on the devices. According to Costa-Orlandi [75] and Pereira et al. [70], in the early stages of biofilm development, basal layers of blastoconidia adhere to the support. During maturation, cells in these layers undergo a morphogenic change that involves filamentation, giving rise to hyphal networks. This process, which can be observed at higher magnification, is a crucial step in the invasion of the surrounding space, whether human tissue or prosthetic device.

## 5. Conclusions

A comparative analysis was conducted between PO and SM LC, considering five key aspects: the biomechanical interaction between the implant and the vertebrae, mechanical behavior, the effect of the implant on the vertebrae, chemical composition of the spacers assessed through Raman spectroscopy, and biocompatibility. Without exception, the PO spacer consistently outperformed the SM spacer. The SM implant exhibits an increase in stiffness beyond 130 kg and subsidence into the system at 150 kg. These effects, however, do not occur in the PO spacer. Although the structural and biomechanical characteristics of PEEK are promising, the surface of the 3D-printed OM PEEK intervertebral spacer requires polishing.

Additionally, strategies to confer antimicrobial properties to these devices must be evaluated to inhibit the development of C. albicans and other microbial biofilms. The biomechanical evaluation of implants is facilitated using the mechanical analysis of prostheses in various case studies, combined with the use of microscopic characterization techniques. Additionally, it offers data that can be utilized to improve prostheses.

## Figures and Tables

**Table 1:** Study cases to perform compressive test analysis in swine’s vertebrae L3–L4.

Study Case	Velocity Displacement	Prosthesis	Stop Criterion
1	2 mm/min	Without LC	Load of 1500 N
2	2 mm/min	Without LC	Load of 2500 N
3	2 mm/min	PO	Load of 3000 N
4	2 mm/min	SM	Load of 3000 N
5	2 mm/min	PO	Load of 3000 N
6	2 mm/min	SM	Load of 3000 N
7	2 mm/min	Without LC	Until material fail
8	2 mm/min	Without LC	Load of 1500 N
9	2 mm/min	Without LC	Until material fail
10	2 mm/min	Without LC	Load of 2000 N
11	2 mm/min	SM	Until material fail
12	2 mm/min	SM	Load of 2000 N
13	2 mm/min	SM	Until material fail
14	2 mm/min	PO	Load of 2000 N
15	2 mm/min	PO	Until material fail
16	2 mm/min	PO	Load of 2000 N

**Table 2:** Wavenumbers and assignments for PEEK [28].

$\mathrm{PEEK}\;∆v\left({cm}^{-1}\right)$		Assignment
97	vw	Phonon ϕ-O-ϕ
135	vw	Phonon ϕ-CO-ϕ
632	w, sh	γCO
646	w	γC−H
669	w	γC−H
680	vw	γC−H
731	vw	γC−H
772	w	γC−H
808	s	γC−H
825	w, sh	γC−H
882	w	γC−H or ring mode
932	w	γC−H, or symmetric νϕ−CO−ϕ
934	vw	γC−H
968	vw	γC−H
1010	vw	Ring stretching mode, or δC−H
1065	vw	γC−H
1096	vw, sh	δϕ
1114	vw	δC−H or νC−O
1146	vs	Symmetric νC−O−C
1161	w, sh	δC−H or ϕ − O and ϕ − CO modes
1173	w, sh	δC−H
1201	m	νϕ−O
1288	w	νϕ−CO−ϕ or ring mode
1307	w	Ring mode
1414	vw	ν−CO−, νC−O−C
1499	vw	Ring stretching mode
1576	w, sh	νC=C
1595	vs	νC=C
1607	s, sh	νC=C
1644	m	νC=O crystalline
1651	m, sh	νC=O amorphous

## Data Availability

The original contributions presented in this study are included in the article. Further inquiries can be directed to the corresponding author.
